# Targeting Host Cell Surface Nucleolin for RSV Therapy: Challenges and Opportunities

**DOI:** 10.3390/vaccines5030027

**Published:** 2017-09-19

**Authors:** Peter Mastrangelo, Michael J. Norris, Wenming Duan, Edward G. Barrett, Theo J. Moraes, Richard G. Hegele

**Affiliations:** 1 Department of Laboratory Medicine and Pathobiology, University of Toronto, Toronto, ON M5S 1A8, Canada; peter.mastrangelo@utoronto.ca (P.M.); mnorris@scripps.edu (M.J.N.); theo.moraes@sickkids.ca (T.J.M.); 2 Department of Paediatrics, The Hospital for Sick Children, Toronto, ON M5G 1L5, Canada; wenming.duan@sickkids.ca; 3 Lovelace Respiratory Research Institute, Albuquerque, NM 87105, USA; tbarrett@lovelacebiomedical.org; 4 Department of Paediatric Laboratory Medicine, The Hospital for Sick Children, Toronto, ON M5G 1L5, Canada

**Keywords:** nucleolin, RSV, virus, therapy

## Abstract

Nucleolin (NCL) has been reported as a cellular receptor for the human respiratory syncytial virus (RSV). We studied the effects of re-purposing AS1411, an anti-cancer compound that binds cell surface NCL, as a possible novel strategy for RSV therapy in vitro and in vivo. AS1411 was administered to RSV-infected cultures of non-polarized (HEp-2) and polarized (MDCK) epithelial cells and to virus-infected mice and cotton rats. Results of in vitro experiments showed that AS1411, used in micromolar concentrations, was associated with decreases in the number of virus-positive cells. Intranasal administration of AS1411 (50 mg/kg) to RSV-infected mice and cotton rats was associated with partial reductions in lung viral titers, decreased virus-associated airway inflammation, and decreased IL-4/IFN-γ ratios when compared to untreated, infected animals. In conclusion, our findings indicate that therapeutic use of AS1411 has modest effects on RSV replication and host response. While the results underscore the challenges of targeting cell surface NCL as a potential novel strategy for RSV therapy, they also highlight the potential of cell surface NCL as a therapeutic target.

## 1. Introduction

The respiratory syncytial virus (RSV, or human orthopneumovirus due to a recent name change [1]), an enveloped, single-stranded RNA *Pneumovirus* which affects all age groups worldwide and causes serious lower respiratory infections such as bronchiolitis and pneumonia [2]. Despite considerable efforts made since the 1960s to develop a safe and effective RSV vaccine, no licensed vaccine is available, and the recent failure of a phase III clinical trial of a vaccine candidate exemplifies the longstanding difficulties experienced in this area [3]. Palivizumab, an antibody that binds to the RSV fusion (F) protein (a viral envelope protein involved in initial viral entry into cells and cell-to-cell spread of infection), is used for RSV prophylaxis in selected “high-risk” patients, and there are reports of RSV resistance developing with respect to this drug [4,5,6,7]. Treatment of RSV infections has predominantly focused on targeting viral components such as the F protein [8] or the polymerase enzyme involved in viral nucleic acid replication [9]. Targeting viral components confers selection pressure for the emergence of drug-resistant viruses [5,10,11].

As obligate intracellular parasites, viruses interact with host cellular components during infection [12,13]. “Targeting the host” is a potential alternative strategy for anti-viral therapy, with this approach conferring a lower probability of the development of viral drug resistance [14]. With our discovery of nucleolin (NCL) as a functional cellular receptor for RSV that putatively interacts with F protein [15], we reasoned that administration of a compound that binds cell surface NCL might be useful for RSV treatment. To test this, we studied the effects of AS1411 (also known as ACT-GRO-777; ACT Corporation, Lexington, KY, USA), a synthetic DNA aptamer that binds cell surface NCL [16,17] and is in the process of phase II clinical trials for cancer treatment [18], on RSV infections in vitro and in vivo. In vitro experiments consisted of AS1411 administration to RSV-infected HEp-2 cells, a non-polarized cell line, or MDCK cells, a polarized cell line that expresses NCL on the apical aspect [19] and models the route of entry of RSV in the respiratory tract [15,20,21,22,23]. In vivo experiments to investigate proof of concept involved RSV challenge, followed by AS1411 administration, in two rodent models: mice [24], and cotton rats [25]. The specific aims of this study were: (1) To determine the effects of AS1411 administration on RSV infection in HEp-2 or MDCK cells; and (2) To determine the effects of AS1411 given therapeutically to RSV-infected mice and cotton rats on levels of infectious virus in the lungs, animal body weight, virus-associated airway inflammation, host IL-4/IFN-γ ratio (an index of Th2 (IL-4), and Th1 (IFN-γ) used in evaluation of new vaccines and drugs [26,27,28]) and bronchoalveolar lavage (BAL) cytology.

## 2. Materials and Methods

### 2.1. General Study Design

**Figure 1:**
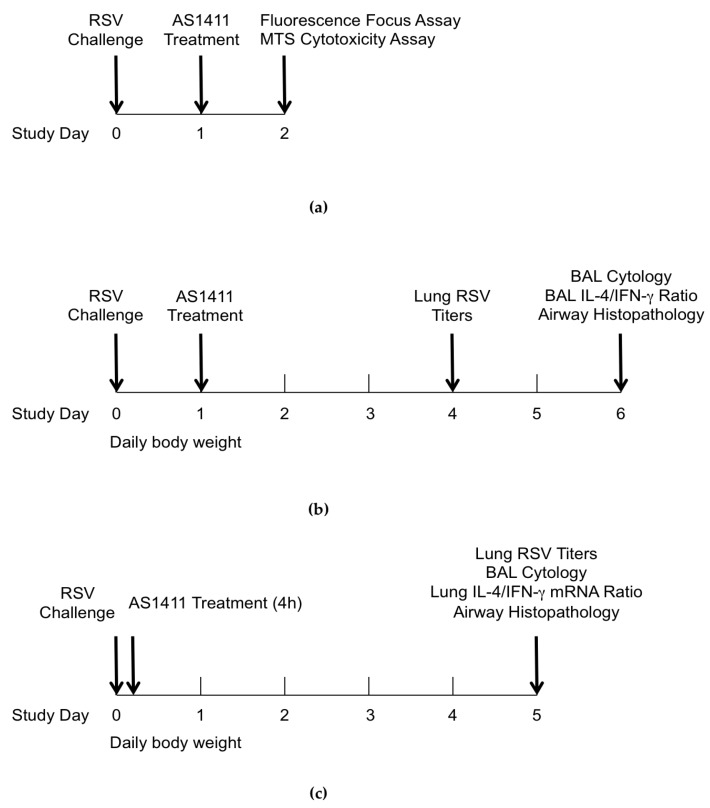
General study design. (**a**) In Vitro experiments with respiratory syncytial virus (RSV)-infected HEp-2 and MDCK cell cultures; (**b**) In Vivo experiments in mouse model; (**c**) In Vivo experiments in cotton rat model. BAL: bronchoalveolar lavage.

Figure 1 shows the schematic outline of the design for in vitro and in vivo experiments. In vitro experiments focused on quantifying HEp-2 or MDCK cells that showed RSV-associated positive immunofluorescence over a range of doses of AS1411. These conditions were chosen to model the in vivo situation in which the virus is not washed off and drug treatment would be given after the virus has already established infection and started spreading to neighboring cells. For in vivo studies, a single dose of AS1411 suspension (50 mg/kg) was delivered intranasally to anesthetized animals. This dose of AS1411 was based on dosing used in humans for cancer treatment, and the intranasal route reflected cell surface NCL being expressed on the apical aspect of airway epithelial cells [20,21].

### 2.2. In Vitro Experiments: Administration of AS1411 to Rsv-Infected HEp-2 Cells or MDCK Cells

HEp-2 cells or MDCK cells, cultured in EMEM (Thermo Fisher Scientific, Waltham, MA, USA) supplemented with 10% fetal bovine serum (FBS) (Thermo Fisher Scientific, Waltham, MA, USA) and antibiotics, were plated onto black, flat, clear-bottomed 96 well plates (Thermo Fisher Scientific, Waltham, MA, USA) (1 × 10^4^ cells/well). The next day RSV A2 (ATCC, Manassas, VA, USA), prepared by centrifugation as previously described [29], was added (multiplicity of infection (MOI)) = 0.1) and incubated for 24 h at 37 °C and 5% CO_2_ to allow at least one full round of virus replication. Twenty-four hours later, AS1411 was applied to the wells in triplicate, starting at 70 μM with serial dilutions down to 4.4 μM. As a negative control we used a cytosine-rich derivative of the same compound (CRO) that does not interact with NCL.

In the fluorescence focus assay (a type of plaque assay adapted from Malhotra et al. [30]), the amount of RSV-infected cells per well is converted to a fluorescent signal. Twenty-four hours after AS1411 or sham treatment, cells were fixed with 75% cold acetone in phosphate-buffered saline (PBS) for 5 min. Following three washes with PBS/0.1% Tween-20 (PBS-Tween) (Thermo Fisher Scientific, Waltham, MA, USA), cells were incubated with a 1:10 dilution of NCL-RSV3 monoclonal antibody pool (Novacastra, Newcastle Upon Tyne, UK) in PBS-Tween/10% FBS for 30 min at room temperature. Cells were washed again with PBS-Tween and incubated with 1:400 dilution (5 μg/mL) of Alexa Fluor 488 anti-mouse IgG (Life Technologies, Carlsbad, CA, USA) in PBS-Tween/10% FBS. Then, after a final wash, fluorescence was detected with a Tecan Infinite M200Pro^TM^ plate reader (excitation: 488 nm; emission: 519 nm) (Tecan Group, Männedorf, Switzerland). Results were expressed as a percentage of fluorescent-positive cells to the corresponding “virus only” control. To assess for potential cytotoxicity, parallel experiments were carried out using the 3-(4,5-dimethylthiazol-2-yl)-5-(3-carboxymethoxyphenyl)-2-(4-sulfophenyl)-2H-tetrazolium (MTS) assay for cell viability (Promega, Madison, WI, USA) according to the manufacturer’s instructions.

### 2.3. Mouse Experiments

Animal studies were approved by the Hospital for Sick Children (Toronto, ON, Canada) Animal Care Committee in accordance with standards of the Canadian Council on Animal Care (Project Approval Number: 34753). Female Balb/c mice, aged 6 to 8 weeks, were purchased from Charles River Laboratories (St. Constant, QC, Canada), kept in a specific pathogen-free environment, and fed food and water ad libitum. The RSV used (RSV-A2, ATCC, Manassas, VA, USA) was propagated in HEp-2 cells and purified over sucrose in an ultracentrifuge [31]. 

For all virus instillation procedures, lightly sedated mice (isoflurane) readily inhaled 50 µL of instillate applied to their nares with a P-200 pipetter while the mouth was held closed. On day 0, mice received 5 × 10^6^ plaque forming units of RSV A2 via intranasal installation (n = 4–6 per group). Mice were monitored and weighed daily.

**Figure 2:**
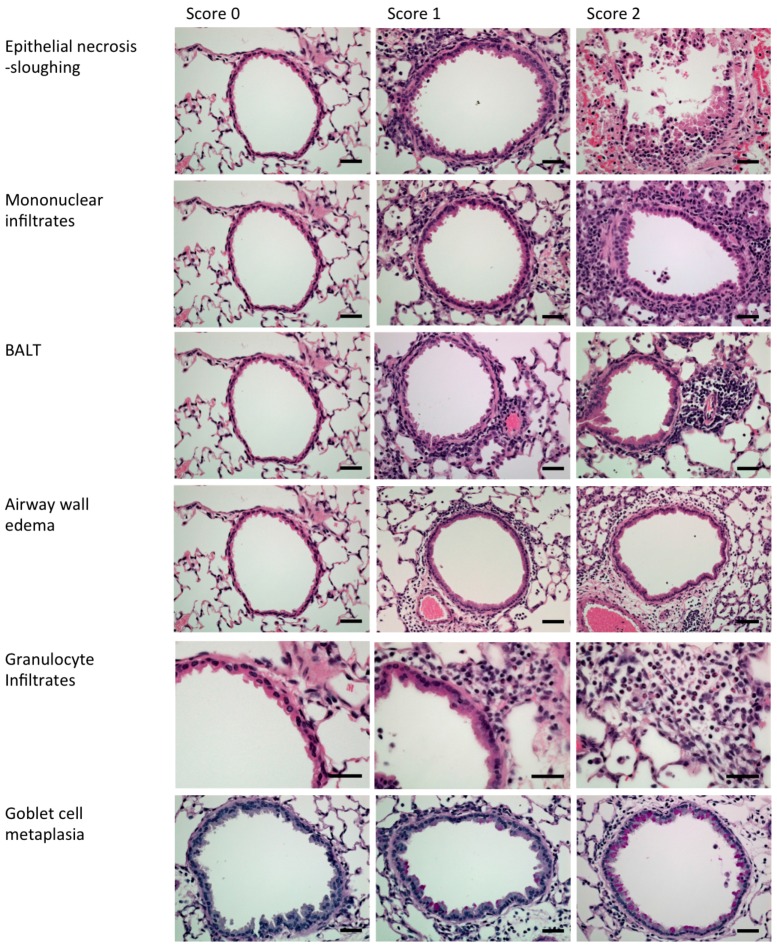
Examples used to generate an airway inflammation histological score in mice. Hematoxylin and eosin staining (top five rows). Periodic acid–Schiff (PAS) staining (bottom row). Scale bars represent 100 μm. BALT: bronchus-associated lymphoid tissue.

On day 1 after RSV challenge, isoflurane-anaesthetized mice received AS1411 (50 mg/kg, diluted in PBS) via intranasal installation. The control group received buffer only. On day 4, when RSV titers were maximal in the mouse model [24], designated mice were euthanized and the lungs removed, weighed and homogenized for viral plaque assays on HEp-2 cell monolayers. On day 6, when RSV-associated airway inflammation is maximal in mice [24], designated animals were euthanized and underwent BAL for enumeration of inflammatory cells and measurement of protein levels of IL-4 and IFN-γ by a commercial assay (Luminex Molecular Diagnostics, Toronto, ON, Canada). Lungs were then removed, inflated and fixed in 10% neutral buffered formalin and processed for preparation of paraffin-embedded blocks. Four-μm-thick sections were put onto glass slides, stained with hematoxylin and eosin (H&E) (Millipore Sigma, Temecula, CA, USA) or periodic acid–Schiff (PAS) (Millipore Sigma, Temecula, CA, USA), and coded. For each animal, an airway inflammation histological score was generated that represented the sum of the following parameters: (1) airway epithelial necrosis/sloughing; (2) mononuclear cell infiltration; (3) granulocyte infiltration; (4) airway wall edema; (5) bronchus-associated lymphoid tissue (BALT) hyperplasia; and (6) goblet cell metaplasia (Figure 2; adapted for mouse from Hegele et al. [32], based on histopathological features of human bronchiolitis [33]).

### 2.4. Cotton Rat Experiments

Four-to-five-week-old cotton rats arrived one week after ordering, were quarantined for 14 days, and were randomized and assigned to treatment or control groups (*n* = 6 animals per group). Animals were maintained in accordance with an approved protocol from the local Institutional Review Board at the Lovelace Respiratory Research Institute (Albuquerque, NM, USA) (Project Approval Number: FY14-107).

On day 0, animals were lightly sedated with isoflurane (Millipore Sigma, Temecula, CA, USA) and received 100 μL containing either 1 × 10^6^ pfu of RSV or uninfected sham inoculum intranasally. Animals were allowed to recover and then 4 h later, cotton rats were again anaesthetized with isoflurane, and received either AS1411 (50 mg/kg) or buffer, administered via intranasal installation. Animals were monitored and weighed daily. On day 5 post-RSV challenge, when lung viral titers in cotton rats are near peak levels and virus-associated lung inflammation can be discerned histologically [25], animals were euthanized and BAL was performed. Lung tissue was then removed and was either weighed and homogenized for viral titers, or snap frozen to isolate RNA, or inflated and fixed with 10% formalin and processed for preparation of paraffin-embedded blocks.

RSV titers were determined by plaque assay of lung homogenates on HEp-2 cells. Lung mRNA cytokine levels for IL-4 and IFN-γ were measured by qPCR. Briefly, RNA was isolated from Trizol-solubilized (Thermo Fisher Scientific, Waltham, MA, USA), snap-frozen lung tissue with the use of the Direct-Zol™ RNA Miniprep kit (#R2053) (Zymo Research Corp., Irvine, CA, USA), according to the manufacturer’s instructions. Cytokine mRNA expression was determined with the use of an ABI TaqMan^®^ RNA-to-C_T_™ 1 Step kit (#4292938) (Thermo Fisher Scientific, Waltham, MA, USA), an ABI 7900HT real-time thermocycler (Applied Biosystems, Foster City, CA, USA) and previously published cytokine-specific primers and probes [34,35]. The forward and reverse primers used for this were: IL-4 Forward: CAT GTA CCG GGA ACT GTA CTC; IL-4 Reverse: GAC ATC CGG TAC AAC CAT CTT; IL-4 Probe: 5HEX/ATG CGA CGA/ZEN/GGA AGC CTT GAA; IFN-Υ Forward: CCA GAC TTT GTA TCA TGG CTT; IFN-γ Reverse: CCG ACA TCT GAG CTA CTT GAA; IFN-γ Probe: 56-FAM/TGC TCT GCT/ZEN/TCT CAC AGG CT. β-Actin was used as the housekeeping gene to normalize samples (ΔCt (Ct target − Ct β-Actin)). mRNA expression was expressed as relative change from baseline (baseline samples = no RSV challenge but treated with vehicle), ΔΔCt (ΔCt animal − avg ΔCt no RSV, vehicle).

For lung histopathological assessment, 4-μm-thick sections were put onto glass slides, stained with H&E or PAS, and coded. For each animal, an airway inflammation score was generated as described above for mice, adapted for cotton rats.

### 2.5. Statistical Analyses

Unless otherwise indicated, we expressed results as mean ± SEM and compared groups by either Mann–Whitney *U* test; Kruskal–Wallis test, or two-way ANOVA with Bonferroni procedure to account for multiple comparisons (PRISM software version 6.0, GraphPad Software Inc., La Jolla, CA, USA, http://www.graphpad.com). We considered a *p* value of ≤0.05 to be statistically significant.

## 3. Results

### 3.1. In Vitro Experiments

**Figure 3:**
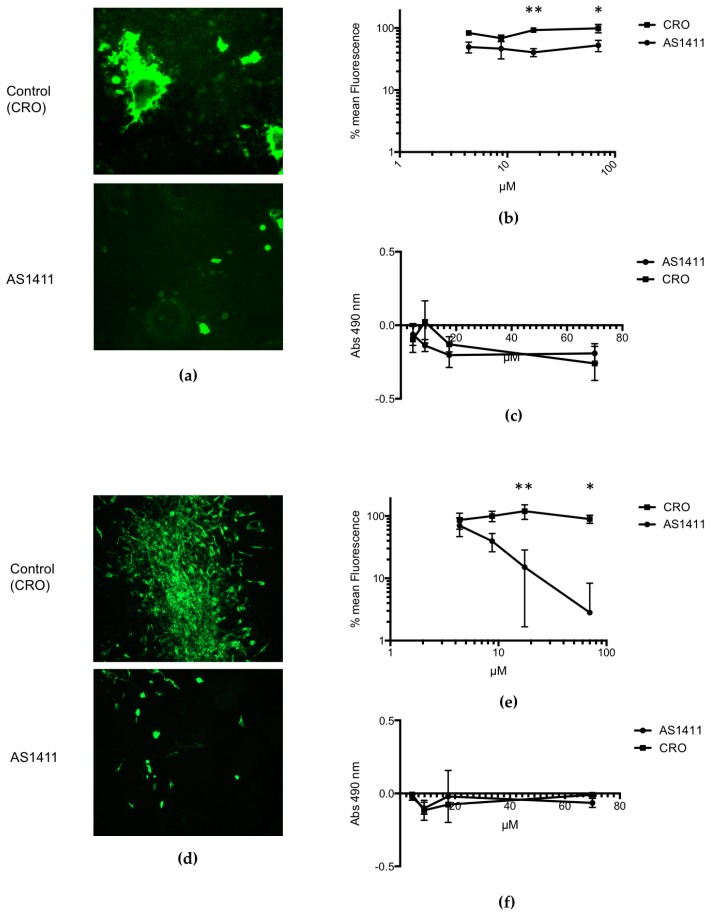
In vitro data demonstrating the efficacy of AS1411 in reducing viral infection. Fluorescence images of HEp-2 cells (**a**) or of MDCK cells (**d**) treated with 17.5 μM AS1411 (bottom) or control (CRO) compound (top), given 24 h after RSV challenge. With AS1411 treatment, the reduction in fluorescence signal is due to reduction in the overall number of RSV-infected cells; HEp-2 cells (**b**) or MDCK cells (**e**) infected with virus (MOI = 0.1) 24 h prior to treating with AS1411/CRO (*n* = 6). A total of 17.5 μM of AS1411 is sufficient to obtain a 60% decrease in RSV fluorescence 24 h after it is added to HEp-2 cells (**b**) (** *p* = 0.0039 and * *p* = 0.01 using Bonferroni procedure; 2-way ANOVA, *p* < 0.0001). Quantities of 17.5 μM and 70 μM of AS1411 are sufficient to obtain a 87% and 97% decrease in RSV fluorescence in MDCK cells (**e**), respectively (** *p* = 0.003 and * *p* = 0.03 respectively using Bonferroni procedure; 2-way ANOVA, *p* < 0.0001). The MTS assay (Promega^TM^, Madison, WI, USA) was performed for both HEp-2 (**c**) and MDCK (**f**) experiments in parallel to assess for cellular toxicity. No difference is seen between AS1411 and CRO in terms of cellular proliferation/health, indicating there was no significant cytotoxicity (2-way ANOVA, with no significant difference between each set of points using Bonferroni procedure).

For RSV-infected HEp-2 cells that were treated with AS1411 or control compound, and analyzed by using a fluorescent focus assay, the results (Figure 3a) demonstrate a moderate reduction in the number of fluorescent cells, i.e., virus-infected cells, associated with AS1411 treatment. Results of dose–response experiments (Figure 3b) showed that even for higher concentrations of AS1411 given (17.5 μM and 70 μM), RSV infection was only reduced about 60% in comparison to controls. However, for RSV-infected MDCK cells that were treated with AS1411 or control compound, and analyzed by using the fluorescent focus assay, the results (Figure 3c) demonstrate a dramatic reduction in the number of fluorescent cells, i.e., virus-infected cells, associated with AS1411 treatment. Results of dose–response experiments (Figure 3d) showed that for higher concentrations of AS1411 given (17.5 μM and 70 μM), RSV infection was reduced on the order of 1–2 logs in comparison to controls. Results of MTS assay, done in parallel, showed that there was no difference in cell viability, i.e., no significant cytotoxicity, associated with AS1411 administration for either HEp-2 or MDCK cells (Figure 3c,f).

### 3.2. Mouse Experiments 

**Figure 4:**
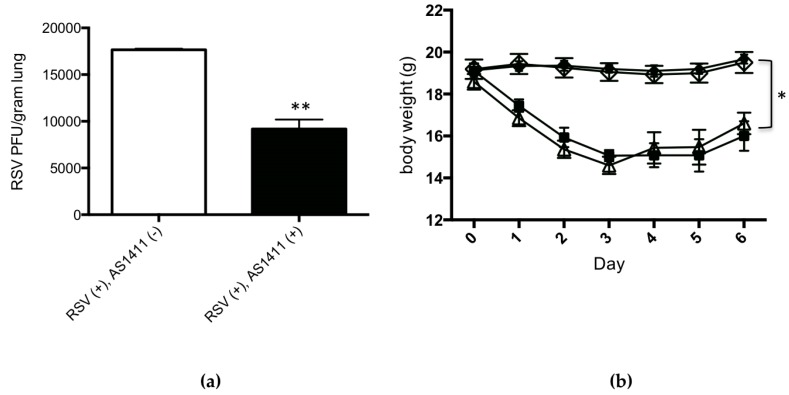

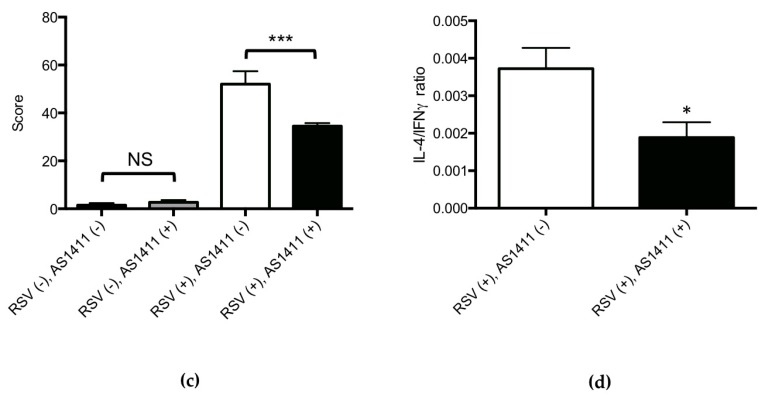
In Vivo mouse data demonstrating the efficacy of AS1411 in reducing viral infection. (**a**) AS1411 (50 mg/kg) given intranasally one day after RSV infection reduced lung virus levels by 0.3 log (~50%) on Day 4 (Mann–Whitney *U* test; ** *p* = 0.03); (**b**) Similar RSV-associated drop and recovery in body weight is obtained in infected animals with or without treatment with AS1411. AS1411 had no effect on body weight in uninfected animals (* *p* < 0.05, 2-way ANOVA with Bonferroni procedure). Significant difference seen between sets of points is due to RSV regardless of presence of absence of AS1411. Square: RSV (+), AS1411 (+); open triangle: RSV (+), AS1411 (−); open diamond: RSV (−), AS1411 (+); circle: RSV (−). AS1411 (−); (**c**) AS1411 treatment decreased RSV-associated airway inflammation (*** *p* = 0.009; Kruskal–Wallis test with Bonferroni procedure) and did not affect airway pathology in uninfected animals (NS = not significant); (**d**) In RSV-infected animals, AS1411 treatment was associated with a significantly decreased IL-4/IFN-γ ratio (* *p* < 0.05; Mann-Whitney *u* test), considered to be a beneficial anti-RSV host response [36,37].

The results of mouse experiments are shown in Figure 4. Concerning viral culture results of lung homogenates (Figure 4a), intranasal administration of AS1411, given 1 day after RSV challenge, resulted in a modest (mean: 0.3 log), statistically significant decrease in lung viral titer on Day 4, in comparison to the sham treatment (*p* = 0.03).

Concerning mouse body weights over the course of the experiment, Figure 4b shows that RSV infection was associated with significant weight loss in comparison to uninfected animals, and this was not affected by AS1411 treatment. In uninfected animals, AS1411 administration did not significantly affect animal body weight.

Histopathological scores of airway inflammation on Day 6 after viral challenge (Figure 4c) were significantly lower in the RSV-infected, AS1411-treated group as compared to the RSV-infected, untreated animals (*p* = 0.009). AS1411 administration to uninfected mice had no significant effects on airway inflammation compared to those animals given sham inoculum.

On Day 6 post-RSV challenge, in BAL fluid specimens (Figure 4d), there was a significant decrease in IL-4/IFN-γ protein levels in RSV-infected, AS1411-treated animals in comparison to the group of infected animals that received sham inoculum (*p* < 0.05). AS1411 administration to uninfected animals did not significantly affect IL-4/IFN-γ ratios in BAL fluid specimens. BAL cell counts were also determined and results are shown in Table 1. RSV challenge was associated with significant increases in BAL total cells, percentage of granulocytes and lymphocytes, and an accompanying significant decrease in percentage of macrophages (*p* < 0.002). These RSV-associated findings were observed irrespective of AS1411 treatment status. Moreover, administration of AS1411 had no significant effect on the total cell counts or percentages of granulocytes, lymphocytes, and macrophages in BAL fluid specimens.

### 3.3. Cotton Rat Experiments

**Figure 5:**
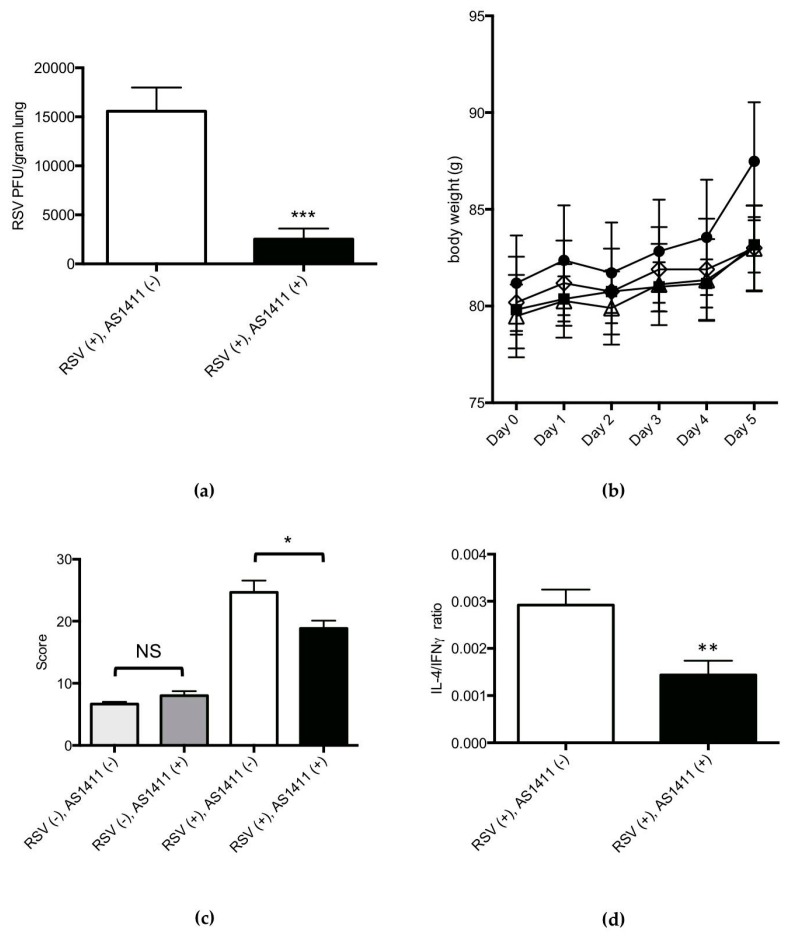
In Vivo cotton rat data demonstrating the efficacy of AS1411 in reducing viral infection. (**a**) AS1411 (50 mg/kg) given intranasally 4 h after RSV infection reduced lung virus levels by 1 log (~90%) on Day 5 (*** *p* = 0.002, Mann–Whitney *U* test); (**b**) Measurement of cotton rat body weights over the course of the experiment. No differences in daily body weights were observed between all experimental groups (*p* > 0.05, 2-way ANOVA). Square: RSV (+), AS1411 (+); open triangle: RSV (+), AS1411 (−); open diamond: RSV (−), AS1411 (+); circle: RSV (−), AS1411 (−); (**c**) AS1411 treatment decreased RSV-associated airway inflammation (* *p* = 0.04, 2-way ANOVA with Bonferroni procedure) and did not affect airway pathology scores in uninfected animals (NS = not significant); (**d**) AS1411 treatment significantly decreased RSV-associated lung tissue IL-4/IFN-γ ratio (** *p* = 0.009, Mann–Whitney *U* test).

Results of cotton rat experiments are shown in Figure 5. Concerning viral cultures (Figure 5a), the group of cotton rats that received AS1411 treatment 4 h after viral challenge had a pronounced (mean: 1 log), statistically significant decrease in lung RSV titer on Day 5 in comparison to RSV-infected, sham-treated animals (*p* = 0.002).

Over the course of the experiment, all animals studied appeared healthy, irrespective of RSV exposure status (as expected with this animal model [25]) or AS1411 treatment status. Cotton rats showed no significant change in body weight in association with RSV infection, irrespective of AS1411 treatment status, over the course of the experiment (Figure 5b).

Histopathological scoring of cotton rat airways (Figure 5c) revealed two main findings: (1) in RSV-infected animals, AS1411 treatment was associated with a significantly decreased airway inflammation score on day 5, in comparison to virus-infected animals that did not receive the compound (*p* = 0.04); (2) administration of AS1411 to uninfected animals did not significantly affect airway inflammation scores, in comparison to the group receiving buffer only. RSV-infected, AS1411-treated animals had a significantly lower IL-4/IFN-γ mRNA ratio than the “RSV only” group (Figure 5d).

For BAL fluid specimens collected on Day 5, the results (Table 1) indicate no significant associations between RSV infection status or AS1411 treatment status on total cell counts, or the percentages of macrophages, lymphocytes or granulocytes.

## 4. Discussion

The results of this study show that AS1411 treatment is effective at reducing levels of RSV observed in three different model systems: HEp-2 cell cultures, MDCK cell cultures, and replicating virus isolated from the lungs of mice and cotton rats. AS1411 administration to HEp-2/MDCK cell cultures was not associated with significant cytotoxicity over the dose range studied. The difference in response between the two cell lines is likely due to the amount of cell surface NCL available and its relative turnover: HEp-2 cells grow rapidly and are non-polarized, while MDCK cells grow more slowly and are polarized with cell surface NCL localized to the apical aspect [19]. In RSV-infected mice and cotton rats, AS1411 administration was associated with decreased airway inflammation and with decreased IL-4/IFN-γ ratios, both of which are considered to reflect a beneficial host response to virus infection. AS1411 administration to uninfected animals did not significantly affect animal body weight or BAL cytology in either species. Overall, these findings support the proof of principle of targeting cell surface NCL for the therapy of RSV infections.

In addition, our results provide further opportunity to extend previous work comparing the mouse and cotton rat models of experimental RSV lung infection [38]. In mouse experiments, AS1411 was given 1 day after inoculation with 5 × 10^6^ pfu of RSV. This interval between virus challenge and drug treatment was associated with a modest (0.3 log) reduction in lung viral titer in AS1411 treated vs. untreated, RSV-infected groups on Day 4, when RSV replication is maximal in the mouse model. Cotton rats challenged with a lower viral inoculum (1 × 10^6^ pfu) and given the same dose of AS1411 at an earlier time-point (4 h) than mice after RSV challenge, showed, as might be predicted, a more profound reduction lung viral titer (1 log) on Day 5. RSV-challenged mice lost approximately 20% of body weight over the course of the experiment, whereas all groups of cotton rats experienced similar degrees of weight gain. Concerning airway histopathology, both species showed significant RSV-associated increases in airway inflammation scores that were improved by AS1411 treatment (Figure 3c and Figure 4c). Of note, baseline airway pathology scores for the uninfected control groups in the mice were near zero (Figure 3c), whereas cotton rats had some very mild baseline airway inflammation (Figure 4c) and higher baseline BAL total cell concentrations (Table 1). Mice, but not cotton rats, showed significant RSV-associated increases in BAL total cell concentrations and changes in the percentages of infiltrating granulocytes, lymphocytes and macrophages (Table 1). These apparent species differences may reflect the use of inbred mice and outbred cotton rats, the latter of which are expected to have greater genetic variability in host inflammatory responses [38]. Irrespective of the differences observed between mice and cotton rats, the effect of AS1411 treatment was similar in both species. In particular, the extent of decrease of RSV-associated airway inflammation with AS1411 treatment (~30–40%) was similar in both species, when compared to virus-infected, untreated animals (Figure 3c and Figure 4c). Further, AS1411-associated decreases in IL-4/IFN-γ ratios were similar in mice and cotton rats (Figure 3d and Figure 4d), even though the cytokines were measured differently: in mice, BAL fluid specimens were tested for IL-4 and IFN-γ protein levels while in cotton rats, mRNA levels in the lungs were measured.

The modest effects observed of treating both mice and cotton rats with relatively large quantities of AS1411 may relate to the fact that the uptake of AS1411 into the cell is not just through the binding and internalization of cell surface NCL but also through other mechanisms [39]. In light of this, it may be advantageous to develop compounds, such as antibodies or other small molecules, that bind and block cell surface NCL primarily and prevent viral fusion. Also, studies in mice and rats with radiolabelled AS1411 show that over 63% AS1411 ends up excreted in the urine within hours of a single intravenous injection [40]. This rapid clearance of AS1411 has meant that, in order to achieve clinically relevant dosing for cancer therapy, regimens such as 7-day continuous intravenous infusions have been required [39]. In light of this last point it seems very likely that further optimization of the dosage regimen of this compound for RSV treatment will be useful.

The mouse and cotton rat experiments involved administration of single doses of AS1411 to establish proof of concept of this therapeutic strategy. Experiments designed to test different doses and dosing regimens of AS1411 can lead to a greater understanding of the effectiveness of targeting NCL in RSV therapy, providing characterization of safety profiles, and determining whether drug resistance can develop through this approach. For RSV, development of drug resistance is a relevant consideration, as instances of resistant viruses have been documented in association with the use of the RSV fusion inhibitor GS-5806 [41], and with palivizumab [4,5,6,7], both of which target the viral F protein.

Interestingly, there are an increasing number of reports indicating that NCL is involved in the replication of a variety of different viruses [42,43,44,45]. In particular, AS1411 has also been shown to inhibit HIV-1 attachment to the host cell [46]. In this work the investigators observed an approximately 60% inhibition of HIV infection using non-polarized cells, similar to our observations in HEp-2 cells, with AS1411 administration in the 0.4 μM to 50 μM range. As such, therapeutics developed around NCL targeting in the host may have broader applications beyond RSV.

Recently, there has been a major resurgence in the area of RSV vaccine development, and promising candidates continue to be developed [47], such that a successful vaccine may be imminent in the next few years. Even so, vaccines will not be useful in cases of acute infection, or in individuals for whom immunization may be contraindicated. Moreover, as has been established for other viruses such as HIV [48] and the hepatitis C virus [49], a multi-drug cocktail may well prove superior to monotherapy for RSV treatment [50]. In conclusion, our current work with AS1411 provides evidence that targeting host NCL is a novel, potentially viable strategy for treating RSV infections.

## Figures and Tables

**Table 1:** BAL cytology for mice and cotton rats ^a^.

		RSV (−), AS1411 (−)	RSV (−), AS1411 (+)	RSV (+) AS1411 (−)	RSV (+), AS1411 (+)	Statistical Analysis ^b^
Mice	Total cell count (×10^4^/mL)	4.47 ± 0.95	5.57 ± 1.18	30.60 ± 11.95	14.48 ± 3.97	S
	% granulocytes	1.45 ± 0.66	2.69 ± 0.63	20.32 ± 7.84	23.31 ± 9.26	S
	% lymphocytes	9.79 ± 2.36	9.28 ± 2.63	44.99 ± 0.18	37.66 ± 9.33	S
	% macrophages	88.72 ± 0.20	88.05 ± 0.19	34.90 ± 0.14	39.00 ± 10.49	S
Cotton rats	Total cell count (×10^5^/mL)	2.96 ± 0.73	4.56 ± 2.17	2.03 ± 1.18	3.63 ± 0.98	NS
	% granulocytes	26.6 ± 12.7	25.14 ± 13.11	21.37 ± 10.80	29.95 ± 13.94	NS
	% lymphocytes	1.48 ± 1.01	0.77 ± 0.56	1.12 ± 0.60	2.60 ± 2.06	NS
	% macrophages	71.88 ± 13.22	74.09 ± 13.57	77.51 ± 10.99	67.45 ± 12.26	NS

^a^ Results displayed as mean ± SEM; ^b^ S: *p* < 0.002 for RSV (+) vs. RSV (−); *p* > 0.05 for AS1411 (+) vs. AS1411 (−) and for the RSV·AS1411 interaction; NS: *p* > 0.05 for RSV (+) vs. RSV (−), AS1411 (+) vs. AS 1411 (−) and for the RSV·AS1411 interaction (2-way ANOVA with Bonferroni procedure).

## References

[1] Adams M.J., Lefkowitz E.J., King A.M.Q., Harrach B., Harrison R.L., Knowles N.J., Kropinski A.M., Krupovic M., Kuhn J.H., Mushegian A.R. (2017). Changes to taxonomy and the International Code of Virus Classification and Nomenclature ratified by the International Committee on Taxonomy of Viruses (2017). Arch. Virol..

[2] Walsh E.E. (2017). Respiratory Syncytial Virus Infection: An Illness for All Ages. Clin. Chest Med..

[3] Novavax RSV F Vaccine Fails Phase III Trial. http://www.genengnews.com/gen-news-highlights/novavax-rsv-f-vaccine-fails-phase-iii-trial/81253207.

[4] Zhu Q., Patel N.K., McAuliffe J.M., Zhu W., Wachter L., McCarthy M.P., Suzich J.A. (2012). Natural polymorphisms and resistance-associated mutations in the fusion protein of respiratory syncytial virus (RSV): Effects on RSV susceptibility to palivizumab. J. Infect. Dis..

[5] Zhu Q., McAuliffe J.M., Patel N.K., Palmer-Hill F.J., Yang C.F., Liang B., Su L., Zhu W., Wachter L., Wilson S. (2011). Analysis of respiratory syncytial virus preclinical and clinical variants resistant to neutralization by monoclonal antibodies palivizumab and/or motavizumab. J. Infect. Dis..

[6] Papenburg J., Carbonneau J., Hamelin M.E., Isabel S., Bouhy X., Ohoumanne N., Dery P., Paes B.A., Corbeil J., Bergeron M.G. (2012). Molecular evolution of respiratory syncytial virus fusion gene, Canada, 2006–2010. Emerg. Infect. Dis..

[7] DeVincenzo J.P., Hall C.B., Kimberlin D.W., Sanchez P.J., Rodriguez W.J., Jantausch B.A., Corey L., Kahn J.S., Englund J.A., Suzich J.A. (2004). Surveillance of clinical isolates of respiratory syncytial virus for palivizumab (Synagis)-resistant mutants. J. Infect. Dis..

[8] Graham B.S., Modjarrad K., McLellan J.S. (2015). Novel antigens for RSV vaccines. Curr. Opin. Immunol..

[9] Fearns R., Deval J. (2016). New antiviral approaches for respiratory syncytial virus and other mononegaviruses: Inhibiting the RNA polymerase. Antiviral. Res..

[10] Battles M.B., Langedijk J.P., Furmanova-Hollenstein P., Chaiwatpongsakorn S., Costello H.M., Kwanten L., Vranckx L., Vink P., Jaensch S., Jonckers T.H. (2016). Molecular mechanism of respiratory syncytial virus fusion inhibitors. Nat. Chem. Biol..

[11] Heylen E., Neyts J., Jochmans D. (2017). Drug candidates and model systems in respiratory syncytial virus antiviral drug discovery. Biochem. Pharmacol..

[12] Shin J., MacCarthy T. (2016). Potential for evolution of complex defense strategies in a multi-scale model of virus-host coevolution. BMC Evol. Biol..

[13] Keener A.B. (2017). Host with the most: Targeting host cells instead of pathogens to fight infectious disease. Nat. Med..

[14] Lee S.M., Yen H.L. (2012). Targeting the host or the virus: Current and novel concepts for antiviral approaches against influenza virus infection. Antiviral. Res..

[15] Tayyari F., Marchant D., Moraes T.J., Duan W., Mastrangelo P., Hegele R.G. (2011). Identification of nucleolin as a cellular receptor for human respiratory syncytial virus. Nat. Med..

[16] Soundararajan S., Chen W., Spicer E.K., Courtenay-Luck N., Fernandes D.J. (2008). The nucleolin targeting aptamer AS1411 destabilizes Bcl-2 messenger RNA in human breast cancer cells. Cancer Res..

[17] Wu J., Song C., Jiang C., Shen X., Qiao Q., Hu Y. (2013). Nucleolin targeting AS1411 modified protein nanoparticle for antitumor drugs delivery. Mol. Pharm..

[18] Rosenberg J.E., Bambury R.M., Van Allen E.M., Drabkin H.A., Lara P.N., Harzstark A.L., Wagle N., Figlin R.A., Smith G.W., Garraway L.A. (2014). A phase II trial of AS1411 (a novel nucleolin-targeted DNA aptamer) in metastatic renal cell carcinoma. Investig. New Drugs.

[19] Balcarova-Stander J., Pfeiffer S.E., Fuller S.D., Simons K. (1984). Development of cell surface polarity in the epithelial Madin-Darby canine kidney (MDCK) cell line. EMBO J..

[20] Shakeri A., Mastrangelo P., Griffin J.K., Moraes T.J., Hegele R.G. (2015). Respiratory syncytial virus receptor expression in the mouse and viral tropism. Histol. Histopathol..

[21] Mastrangelo P., Hegele R.G. (2013). RSV fusion: Time for a new model. Viruses.

[22] Brock S.C., Goldenring J.R., Crowe J.E. (2003). Apical recycling systems regulate directional budding of respiratory syncytial virus from polarized epithelial cells. Proc. Natl. Acad. Sci. USA.

[23] Roberts S.R., Compans R.W., Wertz G.W. (1995). Respiratory syncytial virus matures at the apical surfaces of polarized epithelial cells. J. Virol..

[24] Openshaw P.J. (2013). The mouse model of respiratory syncytial virus disease. Curr. Top. Microbiol. Immunol..

[25] Boukhvalova M.S., Prince G.A., Blanco J.C. (2009). The cotton rat model of respiratory viral infections. Biologicals.

[26] Rivera C.A., Gomez R.S., Diaz R.A., Cespedes P.F., Espinoza J.A., Gonzalez P.A., Riedel C.A., Bueno S.M., Kalergis A.M. (2015). Novel therapies and vaccines against the human respiratory syncytial virus. Expert. Opin. Investig. Drugs.

[27] Feyzi R., Boskabady M.H., Seyedhosseini Tamijani S.M., Rafatpanah H., Rezaei S.A. (2016). The Effect of Safranal on Th1/Th2 Cytokine Balance. Iran. J. Immunol..

[28] Zahzeh M.R., Loukidi B., Meziane W., Haddouche M., Mesli N., Zouaoui Z., Aribi M. (2015). Relationship between NADPH and Th1/Th2 ratio in patients with non- Hodgkin lymphoma who have been exposed to pesticides. J. Blood Med..

[29] Kaan P.M., Hegele R.G. (2003). Interaction between respiratory syncytial virus and particulate matter in guinea pig alveolar macrophages. Am. J. Respir. Cell Mol. Biol..

[30] Malhotra R., Ward M., Bright H., Priest R., Foster M.R., Hurle M., Blair E., Bird M. (2003). Isolation and characterisation of potential respiratory syncytial virus receptor(s) on epithelial cells. Microbes Infect..

[31] Ueba O. (1978). Respiratory syncytial virus. I. Concentration and purification of the infectious virus. Acta Med. Okayama.

[32] Hegele R.G., Robinson P.J., Gonzalez S., Hogg J.C. (1993). Production of acute bronchiolitis in guinea-pigs by human respiratory syncytial virus. Eur. Respir. J..

[33] Aherne W., Bird T., Court S.D., Gardner P.S., McQuillin J. (1970). Pathological changes in virus infections of the lower respiratory tract in children. J. Clin. Pathol..

[34] Blanco J.C., Richardson J.Y., Darnell M.E., Rowzee A., Pletneva L., Porter D.D., Prince G.A. (2002). Cytokine and chemokine gene expression after primary and secondary respiratory syncytial virus infection in cotton rats. J. Infect. Dis..

[35] Blanco J.C., Pletneva L., Boukhvalova M., Richardson J.Y., Harris K.A., Prince G.A. (2004). The cotton rat: An underutilized animal model for human infectious diseases can now be exploited using specific reagents to cytokines, chemokines, and interferons. J. Interferon Cytokine Res..

[36] Becker Y. (2006). Respiratory syncytial virus (RSV) evades the human adaptive immune system by skewing the Th1/Th2 cytokine balance toward increased levels of Th2 cytokines and IgE, markers of allergy—A review. Virus Genes.

[37] Boyoglu-Barnum S., Chirkova T., Todd S.O., Barnum T.R., Gaston K.A., Jorquera P., Haynes L.M., Tripp R.A., Moore M.L., Anderson L.J. (2014). Prophylaxis with a respiratory syncytial virus (RSV) anti-G protein monoclonal antibody shifts the adaptive immune response to RSV rA2-line19F infection from Th2 to Th1 in BALB/c mice. J. Virol..

[38] Vaux-Peretz F., Meignier B. (1990). Comparison of lung histopathology and bronchoalveolar lavage cytology in mice and cotton rats infected with respiratory syncytial virus. Vaccine.

[39] Reyes-Reyes E.M., Teng Y., Bates P.J. (2010). A new paradigm for aptamer therapeutic AS1411 action: Uptake by macropinocytosis and its stimulation by a nucleolin-dependent mechanism. Cancer Res..

[40] Ireson C.R., Kelland L.R. (2006). Discovery and development of anticancer aptamers. Mol. Cancer Ther..

[41] Perron M., Stray K., Kinkade A., Theodore D., Lee G., Eisenberg E., Sangi M., Gilbert B.E., Jordan R., Piedra P.A. (2015). GS-5806 Inhibits a Broad Range of Respiratory Syncytial Virus Clinical Isolates by Blocking the Virus-Cell Fusion Process. Antimicrob. Agents Chemother..

[42] Su P.Y., Wang Y.F., Huang S.W., Lo Y.C., Wang Y.H., Wu S.R., Shieh D.B., Chen S.H., Wang J.R., Lai M.D. (2015). Cell surface nucleolin facilitates enterovirus 71 binding and infection. J. Virol..

[43] Hovanessian A.G. (2006). Midkine, a cytokine that inhibits HIV infection by binding to the cell surface expressed nucleolin. Cell Res..

[44] Thongtan T., Wikan N., Wintachai P., Rattanarungsan C., Srisomsap C., Cheepsunthorn P., Smith D.R. (2012). Characterization of putative Japanese encephalitis virus receptor molecules on microglial cells. J. Med. Virol..

[45] Xue Z., Shan X., Lapeyre B., Melese T. (1993). The amino terminus of mammalian nucleolin specifically recognizes SV40 T-antigen type nuclear localization sequences. Eur. J. Cell Biol..

[46] Perrone R., Butovskaya E., Lago S., Garzino-Demo A., Pannecouque C., Palu G., Richter S.N. (2016). The G-quadruplex-forming aptamer AS1411 potently inhibits HIV-1 attachment to the host cell. Int. J. Antimicrob. Agents.

[47] Stobart C.C., Rostad C.A., Ke Z., Dillard R.S., Hampton C.M., Strauss J.D., Yi H., Hotard A.L., Meng J., Pickles R.J. (2016). A live RSV vaccine with engineered thermostability is immunogenic in cotton rats despite high attenuation. Nat. Commun..

[48] Mbuagbaw L., Mursleen S., Irlam J.H., Spaulding A.B., Rutherford G.W., Siegfried N. (2016). Efavirenz or nevirapine in three-drug combination therapy with two nucleoside or nucleotide-reverse transcriptase inhibitors for initial treatment of HIV infection in antiretroviral-naive individuals. Cochrane Database Syst. Rev..

[49] Kohli A., Shaffer A., Sherman A., Kottilil S. (2014). Treatment of hepatitis C: A systematic review. JAMA.

[50] Simoes E.A., DeVincenzo J.P., Boeckh M., Bont L., Crowe J.E., Griffiths P., Hayden F.G., Hodinka R.L., Smyth R.L., Spencer K. (2015). Challenges and opportunities in developing respiratory syncytial virus therapeutics. J. Infect. Dis..

